# What criteria do decision makers in Thailand use to set priorities for vaccine introduction?

**DOI:** 10.1186/s12889-016-3382-5

**Published:** 2016-08-02

**Authors:** Siriporn Pooripussarakul, Arthorn Riewpaiboon, David Bishai, Charung Muangchana, Sripen Tantivess

**Affiliations:** 1 Department of Pharmacy, Faculty of Pharmacy, Mahidol University, Bangkok, 10400 Thailand; 2 Department of Population, Family and Reproductive Health, Johns Hopkins Bloomberg School of Public Health, Baltimore, MD 21205 USA; 3 National Vaccine Institute, Ministry of Public Health, Nonthaburi, 11000 Thailand; 4 Health Intervention and Technology Assessment Program (HITAP), Ministry of Public Health, Nonthaburi, 11000 Thailand

**Keywords:** Vaccine, Priority-setting, Decision-making

## Abstract

**Background:**

There is a need to identify rational criteria and set priorities for vaccines. In Thailand, many licensed vaccines are being considering for introduction into the Expanded Program on Immunization; thus, the government has to make decisions about which vaccines should be adopted. This study aimed to set priorities for new vaccines and to facilitate decision analysis.

**Methods:**

We used a best-worst scaling study for rank-ordering of vaccines. The candidate vaccines were determined by a set of criteria, including burden of disease, target age group, budget impact, side effect, effectiveness, severity of disease, and cost of vaccine. The criteria were identified from a literature review and by in-depth, open-ended interviews with experts. The priority-setting model was conducted among three groups of stakeholders, including policy makers, healthcare professionals and healthcare administrators. The vaccine data were mapped and then calculated for the probability of selection.

**Results:**

From the candidate vaccines, the probability of hepatitis B vaccine being selected by all respondents (96.67 %) was ranked first. This was followed, respectively, by pneumococcal conjugate vaccine-13 (95.09 %) and *Haemophilus influenzae* type b vaccine (90.87 %). The three groups of stakeholders (policy makers, healthcare professionals and healthcare administrators) showed the same ranking trends. Most severe disease, high fever rate and high disease burden showed the highest coefficients for criterion levels being selected by all respondents. This result can be implied that a vaccine which can prevent most severe disease with high disease burden and has low safety has a greater chance of being selected by respondents in this study.

**Conclusions:**

The priority setting of vaccines through a multiple-criteria approach could contribute to transparency and accountability in the decision-making process. This is a step forward in the development of an evidence-based approach that meets the need of developing country. The methodology is generalizable but its application to another country would require the criteria as relevant to that country.

**Electronic supplementary material:**

The online version of this article (doi:10.1186/s12889-016-3382-5) contains supplementary material, which is available to authorized users.

## Background

Universal vaccination, also known as Expanded Program on Immunization (EPI) or national immunization program (NIP), is generally accepted as a cost-effective intervention for infectious diseases [1]. The World Health Organization (WHO) has recommended EPI vaccines since 1974 [2]. Now WHO recommends routine immunizations for all age groups – children, adolescents and adults—including pneumococcal conjugate vaccine (PCV-13) as well as vaccines against bacillus Calmette–Guérin (BCG), hepatitis B (HepB), polio, diphtheria–tetanus–pertussis (DTP), *Haemophilus influenzae* type b (Hib), rotavirus, measles, rubella and human papilloma virus (HPV) [3, 4]. While new vaccines are available against other diseases, their implementation into the EPI in each country is varied [5, 6]. The Advisory Committee on Immunization Practices (ACIP) or National Immunization Technical Advisory Groups (NITAG) in each country is normally responsible for vaccine selection; however, selection criteria are not well formulated.

Many frameworks have been proposed to guide priority-setting for health interventions, but only a few of them are applicable for vaccines [7, 8]. Vaccines differ in the strength of supporting evidence, the extent of improving health, and economic value to various stakeholders. Vaccines also have special considerations when assessing their cost-effectiveness, including herd immunity, quality of life lost in young children, parental care and productivity lost, nonfinancial economic burden, uncertainty, eradication rate, macroeconomics and tiered pricing [9]. Structured frameworks for vaccine implementation and decision-making have been established by several working groups [6, 8, 10–14]. The criteria may vary in different settings but the contents are likely to have almost the same concerns: for example, disease burden, vaccine characteristics, economic considerations and social concerns. However, these frameworks are lacking in standardization and reproducibility of the evaluation process [8, 12]. The decision-making process may be ad hoc and require judgmental skills [7, 15].

The EPI in Thailand was established in 1977 to eradicate smallpox and to ensure that all children benefit from life-saving vaccines [16]. The current EPI includes vaccines that cover ten diseases: tuberculosis, HepB, diphtheria, tetanus, pertussis, poliomyelitis, measles, mumps, rubella, and Japanese encephalitis (JE). HepB vaccine was the most recent vaccine to be adopted, in 1992. The combination of DTP–HepB vaccine could be integrated into the EPI without any effects on the administration and coverage of EPI vaccines [17]. Currently, there are many licensed vaccines that are being considering for introduction into the EPI: for example, PCV-13, Hib vaccine, rotavirus vaccine, hepatitis A vaccine, inactivated poliomyelitis vaccine, HPV vaccine and varicella zoster vaccine. Factors and evidence considered by the Thai ACIP include policy issues (e.g. public health priority, disease burden, economic issues, vaccine safety and efficacy) and programmatic issues (e.g. strength of the existing EPI and vaccine availability). There is no specific method for developing recommendations by the ACIP. In some cases where data are inadequate, the opinions of ACIP members or other experts are used to make recommendations [16]. The ACIP members may need additional information and critique the opinion of experts until a final consideration is reached.

New vaccine introduction often leads to the financial and programmatic constraints. Decision makers need to take multiple criteria into account simultaneously resulting in risk of informational overload [15]. Thus, there is a need for a multiple-criteria approach which is a concept that allows a trade-off between multiple criteria in a consistent, systematic, and transparent manner [15, 18, 19]. This approach can help identifying the rational criteria for priority setting, weighing their relative importance and also rank-ordering of vaccines [20]. A best-worst scaling (BWS) study on eliciting preferences of new vaccine adoption in Thailand was conducted in 2013 [21]. The BWS study is a quantitative approach that determines the relative importance that respondents assign to various criteria. This method is a criteria-based measure based on the assumption that health interventions, services, products, or policies can be described by these criteria. The BWS method required identification of relevant criteria (representing topic areas) and levels (representing criterion variables, such as category or amount of criteria) for a meaningful study outcome [22]. Respondents’ valuation depends on the criterion level [23]. Respondents are asked to identify the best and the worst (the most/the least preferred or the most/the least important) choices within a series of different scenarios [24]. Each scenario has a same set of criteria and the criterion levels vary across scenarios. It has the ability to estimate the relative importance of all criteria on a common scale [25, 26]. It is also less cognitive and easy to complete the scenarios [25].

The present report follows up on that BWS study, with the aim of setting priorities and facilitating the decision analysis of new vaccines.

## Methods

Setting priority of vaccines included three steps: the BWS study, the assessment of vaccines and the rank-ordering of vaccines.

### BWS study

The criteria and levels were identified from a literature review and by in-depth, open-ended interviews with 11 experts, including policy makers, healthcare providers, healthcare professionals, manufacturers, logisticians and healthcare managers. Seven criteria were identified, including: 1) burden of disease, 2) target age group of vaccine, 3) budget impact, 4) side effect of vaccine, 5) severity of disease, 6) effectiveness and 7) cost of vaccine (see Additional file 1: Table S1). Each criterion had three levels, for example, burden of disease had the level of 10,000, 20,000 and 30,000 new cases per 100,000 population per year, respectively. Main-effects orthogonal design was used to design 18 scenarios, the minimum number necessary to ensure no correlations between the criteria. Each scenario showed seven criteria with levels which represented characteristics of a new vaccine. Before completing the scenario, respondents were informed that a new vaccine in each scenario was adopted and asked them to choose the most and the least important factor of this vaccine. The questionnaire contained 18 scenarios that had to be completed by each respondent.

There were three groups of respondents: policy makers, healthcare professionals and healthcare administrators. Policy makers were defined as being involved with the ACIP, the committee on the National List of Essential Medicines, or the National Health Security Board. Healthcare professionals were defined by membership in an association or royal college of a specific field: for example, pediatrics, obstetrics and gynecology, infectious diseases or preventive medicine. Healthcare practitioners and researchers were also included. Healthcare administrators were those having an administrative role at various levels of the healthcare system: for example, the regional National Health Security Office, the Provincial Public Health Office, or community hospitals.

The questionnaires were administered by postal survey. We also used a snowball technique which the researcher asked for assistance from existing respondents to help identify further respondents with a similar trait of interest. Seventy of 128 questionnaires were completed. Seventy respondents included 11 policy makers, 26 healthcare professionals and 33 healthcare administrators. Then a conditional logistic regression was used to determine coefficients for criterion levels and test for their significance (see Additional file 1: Table S2).

### Assessment of vaccines

We assessed five vaccines, including existing vaccines in the EPI (JE and HepB) and new vaccines (rotavirus, Hib and PCV-13). These vaccines were chosen to illustrate a broad picture of existing vaccines in the EPI and potential vaccines across different disease areas. A systematic review was used to summarize evidence and information of vaccines from all relevant publications. A database search was used to obtain the data of vaccines on seven criteria as identified in the BWS study. We conducted a database search through PubMed, the Cochrane Library, Ovid and Scopus for local evidence of vaccines. Manual searches for additional literature were done via Google and by accessing specific websites of relevant organizations in the country. Budget impacts of vaccines were adjusted to 2015 value [27]. Cost of vaccines were based on the reference prices listed by the Drug and Medical Supply Information Center, Ministry of Public Health, in 2015 [28].

### Rank-ordering of vaccines

The conditional logistic regression allows the estimation of twenty-one coefficients for criterion levels relative to seven criteria. The probability of a vaccine being selected was calculated based on the coefficients of criterion level from the logistic regression models (see Equation 1 and Additional file 1: Table S2):

1 $$ \begin{array}{c}\hfill \mathrm{Logit}\ \left(\mathrm{P}\right) = \ln\ \left[\mathrm{P}/\left(1\hbox{-} \mathrm{P}\right)\right] = {\upbeta}_{1\hbox{-} 3}\mathrm{burden} + {\upbeta}_{4\hbox{-} 6}\mathrm{age} + {\upbeta}_{7\hbox{-} 9}\mathrm{budget} + {\upbeta}_{10\hbox{-} 12}\mathrm{safety} + {\upbeta}_{13\hbox{-} 15}\mathrm{severity}\hfill \\ {}\hfill +{\upbeta}_{16\hbox{-} 18}\mathrm{effectiveness} + {\upbeta}_{19\hbox{-} 21}\mathrm{cost} + \upvarepsilon \hfill \end{array} $$

where P is the probability of a vaccine being selected by respondents, β_i_ (i = 1–21) are the coefficients of the criterion level indicating the probability of selection (for example, β_1–3_ refers to the coefficient of burden-level 1, the coefficient of burden-level 2 and the coefficient of burden-level 3, respectively), and ε is a random error component that varies among scenarios and respondents. The coefficients of criterion level were considered as weights for priority-setting [7, 29]. These weights related to the probability of a vaccine being selected by respondents. Subsequently, the probability of a vaccine being selected with 95 % confidence interval was calculated and rank-ordered.

The calculation of the probability of a vaccine being selected involves several steps. The vaccine data were identified for the level to fit in each criterion. This identification is indicated as ‘1’ or ‘0’ to denote criterion levels of seven criteria. For example, the data of HepB vaccine was identified as ‘1’ for burden-level 1, ‘0’ as burden-level 2, ‘0’ as burden-level 3, ‘1’ for age-level 1, ‘0’ for age-level 2, ‘0’ for age-level 3, ‘0’ for budget-level 1, ‘1’ for budget-level 2, ‘0’ for budget-level 3, ‘0’ for safety-level 1, ‘0’ for safety-level 2, ‘1’ for safety-level 3, ‘0’ for severity-level 1, ‘0’ for severity-level 2, ‘1’ for severity-level 3, ‘0’ for effectiveness-level 1, ‘0’ for effectiveness-level 2, ‘1’ for effectiveness-level 3, ‘0’ for cost-level 1, ‘0’ for cost-level 2 and ‘1’ for cost-level 3. From the regression model of all respondents, the summation of coefficients for HepB vaccine would be 1*(−0.6502) + 1*0.3398 + 1*0.0419 + 1*0.8666 + 1*2.0029 + 1*0.3549 + 1*0.4123 = 3.3682. Then the probability of HepB vaccine being selected by all respondents was calculated as (e^3.3682^/(1 + e^3.3682^))*100 = 96.67 %, where e = 2.71828.

## Results

Table 1 shows vaccine data for Thailand, based on seven criteria developed from the BWS study. All vaccines were administered to children less than 5 years old. Rotavirus infection had the highest burden of disease but lowest severity of disease. PCV-13 had the highest rate of fever. PCV-13, Hib vaccine and rotavirus vaccine had high budget impact due to their high prices, whereas JE vaccine had the lowest budget impact because it is produced domestically.

**Table 1:** Vaccine data for Thailand, based on seven criteria developed from a best–worst scaling (BWS) study

Criterion	Unit	Vaccine				
		JE [27, 28, 37–39]	HepB^a^ [17, 27, 28, 39, 40]	Hib [27, 28, 39, 41, 42]	Rotavirus [27, 28, 39, 43, 44]	PCV-13 [27, 28, 39, 45, 46]
Burden of disease	Annual incidence per 100,000 population	15–18	6,000–8,000	3.8	33,578	11–29
Target group	Age	18–24 months	0–6 months	<5 years	0–5 years	<5 years
Budget impact	Baht per year	239,677,298	355,644,870	1,443,565,660	1,041,994,995	5,121,681,482
Side effect	% of fever	1–2	1–6	2–10	0	33
Severity of disease	n/a	Death (10–20 %); two-thirds of survivors are left with permanent neuropsychiatric sequelae	Hepatocellular carcinoma	Death, disability	Not severe	Invasive pneumococcal disease (meningitis, bacteremia)
Effectiveness	%	84.8	80	95	70–85	89
Cost of vaccine	Baht per course	291	432	1,753	1,265	6,208
Registration year	n/a	2002	2000	1998	2008	2010

^a^This scenario included HepB vaccine at birth and DTP–HepB vaccine at 2, 4 and 6 months, as recommended in the Thai EPI

JE: Japanese encephalitis; HepB: hepatitis B; Hib: *Haemophilus influenzae* type B; PCV-13: pneumococcal conjugate vaccine

Table 2 shows the probability of a vaccine being selected by respondents and rank-ordering of vaccines by groups. From the probability of a vaccine being selected by all respondents, HepB vaccine (96.67 %) was ranked first; it was followed, respectively, by PCV-13 (95.09 %) and Hib vaccine (90.87 %). The three groups of stakeholders (policy makers, healthcare professionals and healthcare administrators) showed the same ranking trends.

**Table 2:** The probability of a vaccine being selected and rank-ordering of vaccines among groups of respondents

Ranking	Policy maker (*n* = 11)		Healthcare professional (*n* = 26)		Healthcare administrator (*n* = 33)		All respondents (*n* = 70)	
	Vaccine	Probability of selection (%) (95 % CI)	Vaccine	Probability of selection (%) (95 % CI)	Vaccine	Probability of selection (%) (95 % CI)	Vaccine	Probability of selection (%) (95 % CI)
1	HepB	91.64 (71.40–97.97)	HepB	95.35 (88.70–98.17)	HepB	96.05 (88.62–98.70)	HepB	96.67 (93.24–98.39)
2	PCV-13	87.24 (60.88–96.78)	PCV-13	93.19 (83.99–97.28)	PCV-13	94.70 (85.13–98.24)	PCV-13	95.09 (90.20–97.60)
3	Hib	78.75 (45.75–94.21)	Hib	81.78 (63.22–92.14)	Hib	91.75 (78.08–97.20)	Hib	90.87 (82.56–95.44)
4	JE	60.29 (25.68–86.96)	JE	73.16 (51.08–87.68)	JE	59.42 (31.92–82.06)	JE	67.80 (50.03–81.59)
5	Rotavirus	33.88 (10.44–69.24)	Rotavirus	27.35 (12.60–49.56)	Rotavirus	52.89 (26.45–77.81)	Rotavirus	33.43 (19.27–51.37)

The regression coefficients of criterion level from the BWS study indicate their relative importance in priority-setting. Due to the different weight of the coefficients, most severe disease, high fever rate and high disease burden showed the highest weight for criterion levels being selected by all respondents (see Additional file 1: Table S2). This result can be implied that a vaccine which can prevent most severe disease with high disease burden and has low safety has a greater chance of being selected by respondents in this study.

## Discussion

The probability of a vaccine being selected was calculated from coefficients of criterion levels of seven criteria (see Equation 1). We considered those coefficients as weights of the probability. HepB vaccine, the first-ranked vaccine, had a high probability of being selected due to its high severity (from hepatocellular carcinoma), high fever rate, high effectiveness, and targeting of infants. This could imply that a new vaccine that is able to protect young children from most severe disease and gain individual health benefits has a higher opportunity to be ranked as a high-priority vaccine. These results were in accordance with other studies that considered burden of disease, effectiveness, safety and cost-effectiveness as major aspects of vaccines [2, 5, 30–32]. However, the impact of immunization should be evaluated through both narrow and broad perspectives. The narrow perspective will focus on cost savings and short-term productivity losses [31], whereas the broad perspective will focus on long-term effects: for example, productivity gains because of improved cognition and education, and behavioral changes because of improved physical health and survival. The broad perspective also includes herd immunity, equity of health outcomes, financial sustainability and macroeconomic impact [4, 12, 33, 34].

The BWS results revealed preferences (a liking for one criterion level over another level in a hypothetical set of criteria) from three groups of respondents. The preferences of policy makers reflected the vaccine policy of the public health system in a societal perspective. The preferences of healthcare professionals reflected clinical practice and self-interest. The preferences of healthcare administrators reflected a provider perspective for the administration and management of vaccines at community, provincial and regional levels. The rank-ordering of vaccines by the three groups showed the same ranking trends (see Table 2). This could imply that all groups may have the same policy stances, and thus reflect the underlying important factor of vaccine adoption for severe disease with high burden. This alignment was in accordance with other studies showing that people may give high priority to a vaccine that protects the population from severe disease with a high burden, but are concerned about the budget impact and safety of that vaccine [10–12].

The criteria and levels included in the BWS study were mapped from a qualitative survey of experts. Criteria from each expert were not the same but were overlapping. This was because each expert had different roles and responsibilities; therefore, his or her concerns were different. However, there were common interests among them: for example, budget impact and price of vaccine were included as the criteria for economic considerations. These criteria were the major concerns of those experts, which was consistent with other studies that considered the same concerns [6, 8, 10–14]. There is no consensus on the number of criteria to include in the BWS study [35]. Criteria may not always include every important aspect to every expert [35]. For a meaningful study outcome, it is important to capture dominant criteria to avoid interferences about omitted criteria. A sufficient wide range of levels should be used to avoid respondents ignoring some criteria because of little difference in levels and to make this criterion more salient to respondents [36]. The suitable number of criteria is context-specific. We tried to balance between what may be important to the experts and what was relevant to the research question and the decision-making context. The discussion with experts and pilot testing was used to assess the importance of criteria and refine as few distinct criteria as possible. Thus well-designed qualitative study that used good sampling of informants could help obtaining the full and broader range of concepts and improving the quality of data.

To our knowledge, no other studies have used the BWS method for rank-ordering of vaccines. Therefore, we cannot directly compare criteria in the regression model. However, they may be compared in terms of components of the criteria. Structured frameworks for vaccine implementation and decision-making have been established by several working groups [6, 8, 10–14]. Those frameworks included disease and vaccine characteristics, economic considerations, feasibility of the program, and qualitative concerns: for example, equity, politics, and social and legal issues. The analytical frameworks are useful in the decision-making process, but only subjectively for prioritization. This study, however, employed the criteria that are amenable for quantification, and also provided prioritization of vaccines. The local evidence on these criteria may also influence evidence-based decision-making in Thailand.

### Limitations

Our study had limitations. First, this study did not include qualitative concerns in the model, e.g. policy, politics, equitability, and social concerns. These data can incorporate difficult or controversial issues that rest heavily on personal judgment and may potentially have a large impact on priority-setting. Second, we used a convenience sample of respondents who were purposively selected and willing to participate in the BWS study. This may not represent the target population. Third, the application of rank-ordering from the same set of criteria may result in high variation of probability of a vaccine being selected. Moreover, this BWS study has been used for the purposes of a student's dissertation. Hence the premise of our study is limited.

### Generalizability

The multiple-criteria approach is generalizable to other settings. However, other countries, with different contexts, may not have the same preferences of criteria: for example, cost of vaccine, vaccine safety, and burden of disease. Then, the criteria may also be different. The prioritization of vaccines should be done with caution when local evidence is limited or applied in a different context. Further application of this approach for prioritization of vaccines in other countries is warranted. More studies are needed to determine the variation of preferences and criteria for new vaccine adoption across countries.

## Conclusions

This study has demonstrated the application of multiple-criteria approach in priority setting process. This is a step forward in the development of an evidence-based approach to set priorities for vaccines that meets the need of developing country. The rank-ordering of vaccines can contribute to transparency and accountability in the decision-making process across various diseases. The methodology is generalizable but its application to another country would require the criteria as relevant to that country.

## Abbreviations

ACIP, Advisory Committee on Immunization Practices; BCG, bacillus Calmette–Guérin; BWS, best–worst scaling; DTP, diphtheria–tetanus–pertussis; EPI, Expanded Program on Immunization; HepB, hepatitis B; Hib, *Haemophilus influenzae* type b; HPV, human papilloma virus; JE, Japanese encephalitis; NIP, national immunization program; NITAG, National Immunization Technical Advisory Groups; PCV-13, pneumococcal conjugate vaccine; WHO, World Health Organization

## Additional file

Additional file 1: Table S1. Definitions of criteria and levels in the BWS study. **Table S2.** The conditional logistic regression results among groups of respondents. (DOCX24 kb)
