# Supplementary material for: What criteria do decision makers in Thailand use to set priorities for vaccine introduction?
Source: BMC Public Health. 2016 Aug 2;16:684. doi: 10.1186/s12889-016-3382-5 (PMC4970258; doi:10.1186/s12889-016-3382-5)
Supplement: Additional file 1: Table S1. — Definitions of criteria and levels in the BWS study. Table S2. The conditional logistic regression results among groups of respondents. (DOCX24 kb) [file 12889_2016_3382_MOESM1_ESM.docx]

**Supplementary Table 1** Definitions of criteria and levels in the BWS study

| Criterion | Definition | Levels |
| --- | --- | --- |
| Burden of disease | Number of new cases per 100,000 population per year affected by the disease or health problem that can be prevented by vaccine | 1. 10,000 new cases per 100,000 population per year |
|  |  | 1. 20,000 new cases per 100,000 population per year |
|  |  | 1. 30,000 new cases per 100,000 population per year |
| Target age group | Target age group for the vaccine | 1. < 5 years |
|  |  | 1. 5–15 years old |
|  |  | 1. > 15 years |
| Budget impact | Financial consequences of adopting the new vaccine | 1. 100 million baht per year |
|  |  | 1. 500 million baht per year |
|  |  | 1. 1,000 million baht per year |
| Side effect | Fever defined by oral temperature of 37.5 °C occurring at 12 hours after vaccination and lasting for 1 day | 1. 10% |
|  |  | 1. 30% |
|  |  | 1. 50% |
| Severity of disease | Symptoms that affect the patient | 1. Not severe: mild disease that can be successfully treated |
|  |  | 1. Moderately severe: disease affecting normal life and requiring treatment, but not affecting long-term health |
|  |  | 1. Most severe: disease causing death, permanent disability, or long-term effects that require continuity of treatment |
| Effectiveness | The percentage reduction of disease provided by vaccine | 1. 60% |
|  |  | 1. 70% |
|  |  | 1. 80% |
| Cost of vaccine | Price of vaccine per course of vaccination | 1. 100 baht |
|  |  | 1. 300 baht |
|  |  | 1. 500 baht |

**Supplementary Table 2** The conditional logistic regression results among groups of respondents

| Criterion levels | Policy maker (*n* = 11) | | Health professional (*n* = 26) | | Health administrator (*n* = 33) | | All respondents (*n* = 70) | |
| --- | --- | --- | --- | --- | --- | --- | --- | --- |
|  | Coefficient (95% CI) | *p-*value | Coefficient (95% CI) | *p-*value | Coefficient (95% CI) | *p-*value | Coefficient (95% CI) | *p-*value |
| Burden of disease |  |  |  |  |  |  |  |  |
| 10,000 cases per year | -0.645(-1.074,-0.215) | 0.003 | -0.667(-0.964,-0.369) | 0.000 | -0.583(-0.787,-0.273) | 0.000 | -0.650(-0.856,-0.445) | 0.000 |
| 20,000 cases per year | 0.079(-0.177,0.335) | 0.55 | -0.108(-0.283,0.067) | 0.23 | 0.025(-0.136,0.187) | 0.76 | -0.017(-.140,0.107) | 0.79 |
| 30,000 cases per year | 0.566(0.132,1.000) | 0.01 | 0.774(0.426,1.123) | 0.000 | 0.504(0.222,0.787) | 0.001 | 0.667(0.446,0.887) | 0.000 |
| Target age group |  |  |  |  |  |  |  |  |
| < 5 years | 0.109(-0.291,0.509) | 0.59 | 0.388(0.148,0.629) | 0.002 | 0.322(0.104,0.540) | 0.004 | 0.340(0.168,0.512) | 0.000 |
| 5–15 years old | -0.031(-0.231,0.168) | 0.76 | -0.011(-0.143,0.122) | 0.88 | -0.231(-0.392,-0.069) | 0.005 | -0.126(-0.242,-0.011) | 0.03 |
| > 15 years | -0.078(-0.442,0.286) | 0.68 | -0.378(-0.628,-0.128) | 0.003 | -0.092(-0.340,0.157) | 0.47 | -0.213(-0.388,-0.039) | 0.02 |
| Budget impact |  |  |  |  |  |  |  |  |
| 100 million baht per year | -0.392(-1.228,0.444) | 0.36 | -0.526(-0.894,-0.157) | 0.005 | -0.709(-1.014,-0.403) | 0.000 | -0.647(-0.880,-0.413) | 0.000 |
| 500 million baht per year | -0.196(-0.527,0.135) | 0.25 | 0.080(-0.110,0.270) | 0.41 | 0.080(-0.081,0.241) | 0.33 | 0.0420(-0.081,0.165) | 0.51 |
| 1,000 million baht per year | 0.588(-0.234,1.410) | 0.16 | 0.446(0.070,0.822) | 0.02 | 0.628(0.287,0.970) | 0.000 | 0.605(0.348,0.862) | 0.000 |
| Side effect |  |  |  |  |  |  |  |  |
| 10% | -0.827(-1.294,-0.361) | 0.001 | -0.756(-1.109,-0.403) | 0.000 | -0.602(-0.896,-0.307) | 0.000 | -0.766(-0.981,-0.551) | 0.000 |
| 30% | -0.215(-0.401,-0.028) | 0.02 | -0.007(-0.220,0.207) | 0.95 | -0.127(-0.304,0.050) | 0.16 | -0.101(-0.245,0.043) | 0.17 |
| 50% | 1.042(0.539,1.544) | 0.000 | 0.763(0.352,1.173) | 0.000 | 0.726(0.384,1.072) | 0.000 | 0.867(0.613,1.121) | 0.000 |
| Severity of disease |  |  |  |  |  |  |  |  |
| Not severe disease | -1.181(-1.646,-0.716) | 0.000 | -1.492(-1.772,-1.213) | 0.000 | -1.218(-1.643,-0.793) | 0.000 | -1.500(-1.735,-1.265) | 0.000 |
| Moderately severe disease | -0.500(-0.818,-0.182) | 0.002 | -0.406(-0.637,-0.174) | 0.001 | -0.451(-0.692,-0.209) | 0.000 | -0.503(-0.690,-0.316) | 0.000 |
| Most severe disease | 1.681(1.148,2.213) | 0.000 | 1.898(1.573,2.223) | 0.000 | 1.668(1.235,2.101) | 0.00 | 2.003(1.717,2.288) | 0.000 |
| Effectiveness |  |  |  |  |  |  |  |  |
| 60% | -0.369(-0.852,0.115) | 0.14 | -0.291(-0.645,0.063) | 0.11 | -0.081(-0.473,0.310) | 0.68 | -0.223(-0.472,0.026) | 0.08 |
| 70% | 0.020(-0.210,0.251) | 0.86 | -0.119(-0.351,0.113) | 0.32 | -0.180(-0.372,0.012) | 0.07 | -0.132(-0.253,-0.011) | 0.03 |
| 80% | 0.348(-0.121,0.817) | 0.15 | 0.410(0.031,0.789) | 0.03 | 0.262(-0.141,0.664) | 0.20 | 0.355(0.101,0.609) | 0.001 |
| Cost of vaccine |  |  |  |  |  |  |  |  |
| 100 baht per course | -0.200(-0.831,0.430) | 0.53 | -0.403(-0.753,-0.053) | 0.02 | -0.630(-0.989,-0.271) | 0.001 | -0.522(-0.764,-0.281) | 0.000 |
| 300 baht per course | 0.144(-0.283,0.571) | 0.51 | 0.255(0.045,0.465) | 0.02 | -0.030(-0.241,0.180) | 0.78 | 0.110(-0.033,0.253) | 0.13 |
| 500 baht per course | 0.056(-0.596,0.708) | 0.87 | 0.148(-0.227,0.523) | 0.44 | 0.660(0.301,1.020) | 0.000 | 0.412(0.159,0.666) | 0.001 |
| Log pseudolikelihood | -682.0672 | | -1,583.7241 | | -2,048.5286 | | -5,148.7371 | |
| Pseudo *R*^2^ | 0.0784 | | 0.0946 | | 0.0773 | | 0.0759 | |
